# Young apple tree development under agroforestry radiative conditions: a multi-scale morphological and architectural dataset

**DOI:** 10.1093/aobpla/plaf029

**Published:** 2025-06-12

**Authors:** Francesco Reyes, Benjamin Pitchers, Christophe Pradal, Pierre-Éric Lauri

**Affiliations:** DSV, University of Studies of Modena and Reggio Emilia, Reggio Emilia, Italy; ABSys, University of Montpellier, CIHEAM-IAMM, CIRAD, INRAE, Institut Agro, Montpellier, France; CIRAD, UMR AGAP Institut, Montpellier 34398, France; AGAP Institut, University of Montpellier, CIRAD, INRAE, Institut Agro, Montpellier, France; Inria and LIRMM, University of Montpellier, CNRS, Montpellier, France; ABSys, University of Montpellier, CIHEAM-IAMM, CIRAD, INRAE, Institut Agro, Montpellier, France; Plants, Ecosystems & Climate

**Keywords:** agroforestry, fruit tree, growing conditions, light, MTG, multi-scale tree graph, OpenAlea, tree architecture

## Abstract

Agroforestry is a major adaptation and mitigation strategy facing climate warming, but its agronomic viability depends on actual plant responses to shade conditions. Growing fruit trees under dominant trees may reduce the risks related to extreme climatic events, such as frost or heat waves. Nonetheless, except for some sciaphilous plants, such as coffee or cacao, their physiological and architectural responses to agroforestry conditions are little known, especially in temperate climate. We present a dataset describing the architecture and morphology of 45 young apple trees, acquired in two consecutive years, along a radiative gradient, as in three growing conditions of an agroforestry plot: (i) the open field, (ii) between, and (iii) along rows of dominant walnut trees. The data are stored as standard multi-scale tree graphs that allow to store the topology, geometry, and attributes of the plant at different scales. It includes plant traits at three topological scales: whole tree, growth unit, and the internode. The traits include organ fate (latent, vegetative, floral bud, and bud extinction sites); length and an estimate of the leaf area of growth units; diameter, zenith, and azimuth angles of second-order branches. The number of leaves, flowers, fruits, and fruit drops is also counted on a sample of 10, possibly apical, flower buds per tree. The dataset includes ancillary measurements on sampled shoots, used to derive allometric relationships between shoot length and leaf area; and an estimate of the radiation reaching each apple tree during the vegetative season. The multi-scale description and the different light growing conditions characterizing the digitized trees allow to investigate relationships between the shade-related agroforestry environment and the apple tree morphological and architectural plasticity, during the early tree development, from the internode to the whole tree.

## Teaser

The dataset portrays the architectural growth of 45 apple trees developing in different illumination conditions, determined by the various shading imposed by the presence/absence of nearby dominant walnut trees in an agroforestry environment. Apple tree morphological characteristics (e.g. the leaf area, distance between leaves, the branch inclination, ...) are described as seen at different spatial resolution, namely considering thair average for the whole plant or as they vary within single branches.

## Introduction

Agroforestry combines adaptation and mitigation strategies facing climate warming (Quinkenstein et al. 2009, Garrity 2012, Hallema et al. 2014, Jacobs et al. 2022), but its agronomic viability depends on the plant responses to the facilitation and competition mechanisms emerging in multi-layer plant communities (Swieter et al. 2022). Growing fruit trees as a secondary layer may milden the effects of extreme climatic events, such as frost or heat waves, and risks such as flower loss, and thermal and drought stresses (Reyes et al. 2021, Jacobs et al. 2022). Nonetheless, the reduced light availability impacts carbon assimilation, while its spectral composition may trigger shade avoidance and/or adaptation responses based on modifications of organs’ morphological traits (Smith and Whitelam 1997, Lauri and Gautier 2024). Overall, the plant growth and structure may be importantly affected. Nonetheless, knowledge on these responses is limited, especially in temperate perennial species (Lauri 2021, Pitchers et al. 2021).

### Data description

**Figure 1.**
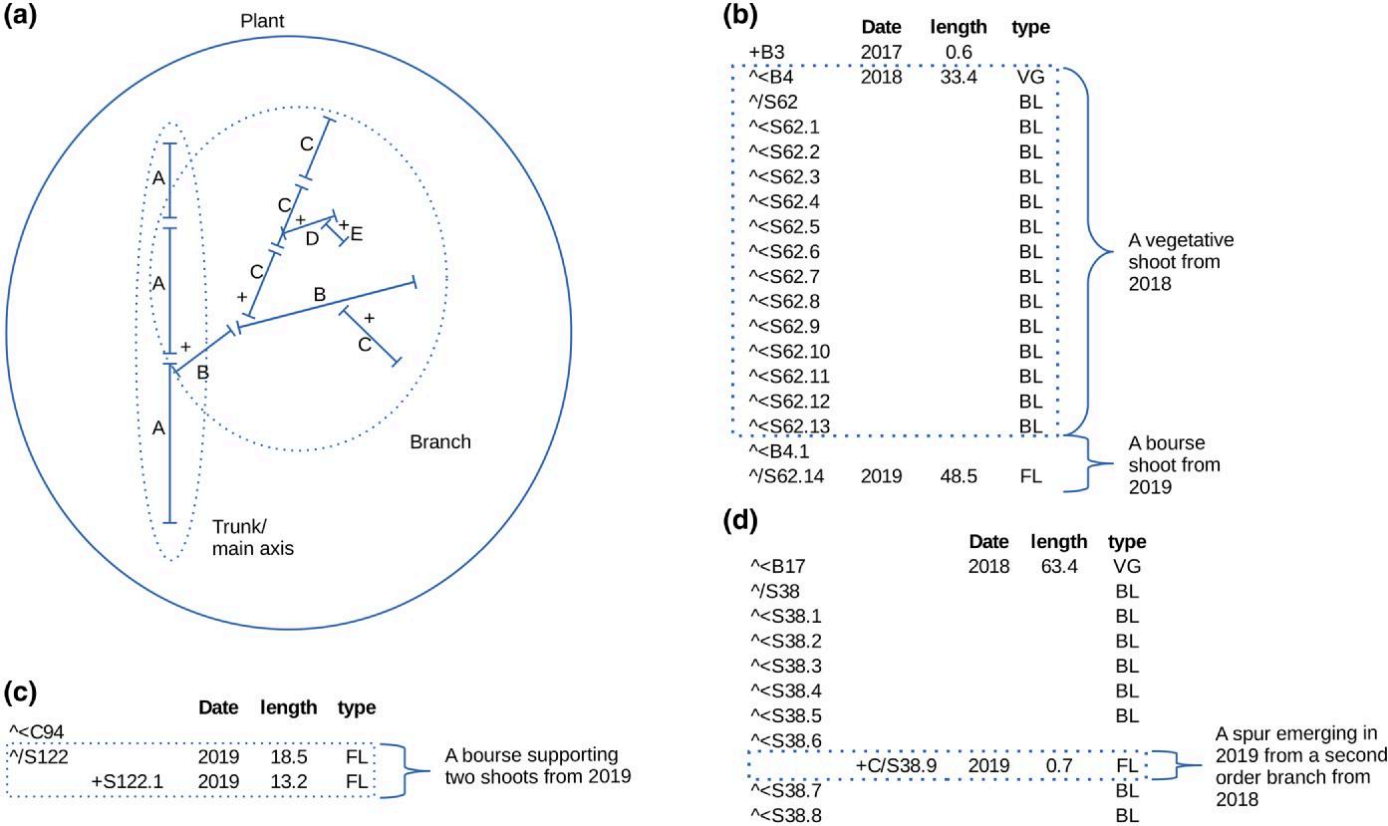
Multi-scale organization of the plant architecture (a) and examples of its coding (b–d). (a) The plant and GUs of 1st-A, 2nd-B, 3rd-C, 4th-D, and 5th-E orders are explicitly described in the MTG (solid lines); branches and the main axis may be inferred by simple topological analysis of the MTG (dotted lines). The internode scale (S) is not shown. (b–d) MTG coding of characteristic apple tree structures: (b) a second-order ‘B’ vegetative shoot (sequence of successive internodes ‘<S’ on the same year 2018) with latent buds ‘BL’, followed by a bourse shoot in the following year (defined by a successive GU ‘<B’ composed of an element from 2019 ‘/S’, which is carrying a floral bud ‘FL’); (c) a double bourse shoot (a bourse with two shoots: one shoot ‘/S122’ of length 18.5 cm following the previous element (not shown) and carrying an apical floral bud ‘FL’, and a second shoot ‘S122.1’ of length 13.2 cm, branching ‘+’ from the first element and also carrying a floral bud ‘FL’), (d) a third-order spur ‘C’ of length 0.7 cm, branching ‘+’ from the internode ‘S38.6’, which belongs to the second-order branch ‘B17’, and carrying a floral bud ‘FL’.

To shed further light on architectural and morphological perennial plant responses to variable shade conditions, we present a dataset describing the architecture and morphology of a large sample (*n* = 45) of apple trees during their third and fourth years of growth (2018 and 2019, respectively). Trees are cultivated in three growing conditions: along the row (AFR) and the inter-row (AFIR) of an intercropping field, under dominant walnut trees, and in the open field (AC). The topology of each tree is described at three topological scales (whole plant, P; growth unit, GU, as being part of either a first- to fifth-order branch; and internode, S) by means of a multi-scale tree graph (MTG) (Godin and Caraglio 1998, Godin 1999, Pradal and Godin 2020) under the OpenAlea platform (Pradal et al. 2008; Fig. 1). The characterization includes several variables depending on the considered scale (SI Multiscale organization of plant entities.ods).

**Figure 2.**
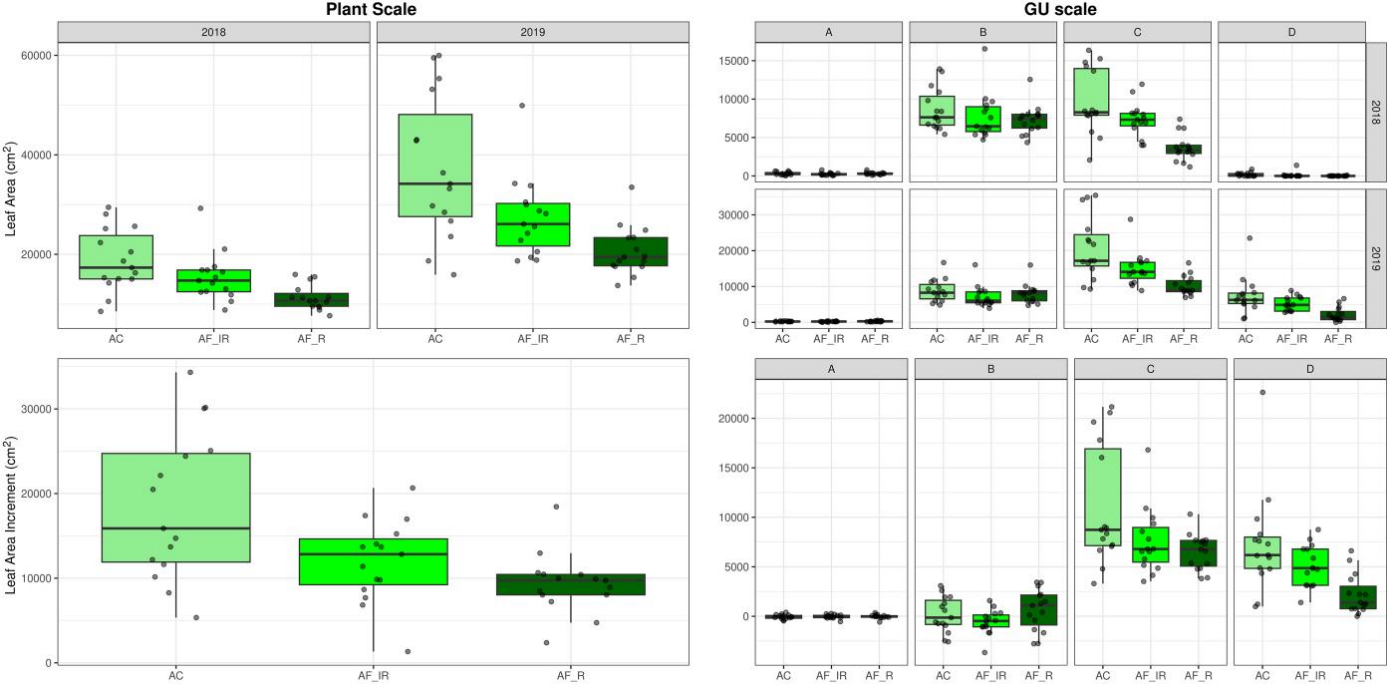
Leaf area and leaf area increment at plant and GU scales in years 2018 and 2019. Leaf area values at a given scale and year are calculated as the sum of leaf areas present on the same plant at that scale. Leaf area increments are calculated as difference in the leaf area estimated for a certain scale in the year 2019 minus the value obtained at the same scale in the year 2018. A: 1st, B: 2nd, C: 3rd, D: 4th orders GUs. AC, in the open field; AFIR, along the inter-row; AFR, along the row of the intercropping field.

At plant scale, the total leaf area is provided (Fig. 2A). GUs of all orders are provided with length, basal and apical diameters, and an estimate of leaf area based on allometric relationships with shoot length (for flowering shoots, including and excluding the bourse, if present; Fig. 2B). The height of the grafting point is given for the main axis; azimuth and zenith angles for the first GU of second-order branches; the number of leaves, flowers, fruits, and fruit drops for up to 10, possibly terminal, floral units per tree. The fate of single buds is distinguished into floral, vegetative, latent, or extinction (shoot death).

### Additional measurements

Two spreadsheets provide (i) the growing conditions (AC, AFIR, and AFR), and an estimate of the total PAR light availability during the growing season for each tree in 2018 (SI Tree PAR light conditions.csv; Pitchers et al. 2021); (ii) lengths and leaf areas of sampled GUs used to derive allometric relationships and infer the leaf areas of the digitized trees, depending on light conditions and shoot type (bourse and vegetative shoots; SI Allometric relationships.ods).

Additionally, data extracted from the MTGs concerning multiple topological scales is also provided in table format. In particular, for every tree, the number of GUs and the total leaf area, depending on the year (SI tree.csv); for every GU, its ramification order, leaf area and number of leaves, flowers, fruit, and supported GUs (SI GU.scv); and for each first-order branch, the insertion angle (SI B_diam.csv) and base diameter (SI B_angle.csv).

### Data value for research

The presented dataset is the first one portraying a large sample of digitized trees growing as an intermediate layer in agroforestry conditions and in the open field.

The dataset is of high interest for studies concerning the plasticity of early development perennial plants to various degrees of shade.

The information on the vegetative and reproductive growth are also of interest for agroforestry counsellors and practitioners, who may search for indications of the apple tree’s potential growth when growing as an intermediate layer, depending on light availability and position in the field, when designing an agroforestry system.

The multi-scale characteristic of the dataset may allow to examine the data not only at the different provided topological scales but also at scales derived from the original ones.

## Materials and methods

### Study site

A walnut tree (*Juglans nigra* × *Juglans regia* NG23) plot was first planted in 1995, in the Domaine Départemental de Restinclières (Prades-le-Lez, Hérault, southern France, 43° 42′ 12.168″ N, 3° 51′ 29.872″ E). The intra-row and inter-row distances were 4 and 13 m, respectively, for a total of 25 trees, while rows had an east/west orientation. A legume (*Medicago sativa* L.) was used for soil cover, and trees were trained for timber production by pruning the lowest branches and thinning the smallest ones in 2007. A total of 150 apple trees (*Malus domestica* Borkh. ‘Dalinette’ grafted on Geneva® G202 C.O.V. rootstock) were added to the plot in 2016, according to three main growing conditions: along and between the walnut tree rows, thus creating a secondary tree layer, and further (≥15 m) from the walnut trees in an open field. Considering both tree species together, the between-tree row distance became 6.5 m and the within-row distance 1.3 m. Apple trees alone covered an area of about 1300 m^2^ while, considering also the walnut trees, the total area was ∼2400 m^2^. Apple trees were left unpruned, while fruitlets were entirely removed in years 2017 and 2018, to avoid inhibiting the vegetative growth in the same year and the fruit set in the following year.

### Tree architecture

Apple tree trunk cross-sectional area was measured for all apple trees in 2018. This became the basis for the selection of 45 trees, 15 per growing condition, covering a wide gradient of trunk dimensions.

The architectures of individual trees were first acquired by the end of the growing season in 2018. Plants were numbered, and their structures described at three scales: the whole plant (P), the GU, and single (for observations prior to 2019) or groups (for observations in 2019) of internodes and buds (S). A plant part described at a relatively coarse scale (whole plant or GU) may be decomposed (‘/’) into finer scale topological elements (GUs or S, respectively).

The plant description started with the measurement of the height of the grafting point. Then, starting from the trunk bottom (or grafting point) and moving upward, following individual ramifications, visual observation allowed to identify the basis and apex of GUs from years 2016, 2017, and 2018. The topological connections of individual GUs were distinguished in successions (‘<’) and branchings (‘+’) (Godin and Caraglio 1998). Basal and apical diameters of individual GUs were measured with a digital calliper, while their length was measured with a tape measure. When a floral structure was present, its length was also noted. GUs were named according to the order of ramification to which they belonged: first (A), second (B), third (C), fourth (D), and fifth (E) order axes. Concerning the second-order branches, their azimuth and zenith angles at their insertion point into the main (or first order) axis were measured with a protractor. The ramification order later allowed for checking topological consistency in a process of data curation.

Considering the buds present along the tree structure, their fate in years 2018 and 2019 was distinguished into four classes (‘type’ variable): as giving origin to a floral (FL; including a bourse and possibly a bourse shoot) or vegetative (VG) GU, or remaining a latent bud (BL); in addition, the mortality of a previously existing axis was noted as an extinction (EX; see Lauri et al. 1995).

In spring 2019, up to 10, possibly apical, flower buds per tree were tagged, and their number of leaves, flowers, and fruits were counted. Then, the number of fruits on tagged flower buds was counted again in late September 2019, to derive the number of fruit drops. All buds that did not generate flowers or shoots, thus remaining dormant, were confirmed as latent buds. The same groups of internodes (S) measured in both 2018 and 2019 were noted with a ‘*’ symbol in a new line in the MTG in 2019, indicating that they represent the same entity (S) as in 2018, but with different attributes in 2019.

In some cases, describing the same variable at multiple scales or plant elements would be redundant, and it was preferred to populate it only once. This is the case for, e.g. the base diameter of a GU and the apical diameter of its parent GU, or the date of a GU and the ones of its internodes.

Some of the structural variables were occasionally not measured due to constraints potentially impacting their accuracy. In particular, (i) length and diameter of some particularly small GUs and bourses; concerning second-order branches, (ii) angle, in branches particularly short and difficult to reach which, together, made it impossible to obtain an accurate measure using a protractor; (iii) azimuth, for branches so high in the canopy (from the year 2019) that measuring would require important bending of the tree structure. Finally, as a consequence of branch breaking due to mechanical accidents during field operations, minor parts of a branching structure may occasionally be longer in 2018 than in 2019.

### Allometric relationships

Upon a visual observation, AC and AFIR trees presented similar shoot morphology, suggesting a similar leaf area to shoot length ratio, while this differed in AFR trees. Based on this, allometric relationships were drawn for AC and AFR trees, whereas the first was later used to infer leaf area on AC and AFIR trees, while the second on AFIR trees. In particular, 30 vegetative GU and 30 fully grown bourse shoots were sampled from ancillary nearby AC and AFR trees in 2018. Their lengths were measured with a tape measure, and leaf area scanned and measured via the WinFOLIA software (Regent Instrument). Allometric relationships between shoot length and leaf area were determined depending on shoot type (vegetative, bourse shoot) and light conditions and later used to infer the leaf area of individual GUs described in the MTGs.

### Light environment

The radiation reaching each apple tree was characterized starting from hemispherical photographs (Sony NEX7 DSLR camera with a lens from Regent Instrument Inc., Québec, Canada). Photographs were taken above each apple tree, twice during the year 2018 (before walnut leaf emergence and at their maximum leaf area development), ∼1 h before sunrise in diffuse light conditions. Images were analysed with the WinSCANOPY™ software (Regent Instruments Inc.) to draw the suntrack during the days of photo acquisition. These were then used within the software to infer an estimate of the photosynthetically active radiation (PAR) received by each apple tree for the two walnut phenological phases considered by the photographs. The quantity of light received by the apple trees on each day was then estimated by linear interpolation between the two dates. Total incident PAR values for the vegetative season were obtained by summing their daily values.

## Supplementary Material

plaf029_Supplementary_Data

## Data Availability

The multi-scale tree graphs are permanently available on a https://github.com/openalea/GAFAM repository and are assigned a permanent doi on https://zenodo.org/records/14422003. The spreadsheet tables of variables extracted from MTGs are provided as Supplementary data of this article.
