# Supplementary material for: Young apple tree development under agroforestry radiative conditions: a multi-scale morphological and architectural dataset
Source: AoB Plants. 2025 Jun 12;17(4):plaf029. doi: 10.1093/aobpla/plaf029 (PMC12310329; doi:10.1093/aobpla/plaf029)
Supplement: plaf029_Supplementary_Data [file plaf029_supplementary_data.zip › Supporting Information - MTGs FAQs and Supporting Information - Multiscale organization of plant entities.pdf]

Supporting Information - Frequently Asked Questions on the MTGs

Why do we always find series of just 1 internode with date 2019?
The “S” scale represents single internodes in observations from 2018 and groups of internodes (possibly GUs) in observations from 2019.

How is that the main axis is composed of multiple successive main axis“A” metamers?
The elements of intermediate scales (B, C, D) are growth units, so that successive A indicate sections of the main axis that grew one following the previous one.

What the 6 variables for diameter (diameter, diameter_b, diameter_a, diameter_2018, diameter_b2018, diameter_a2018) represent ?
Variables including “2018” are referred to measurements made in 2018; the others refer to measurements made in 2019.
Variables including “diameter_b” refer to basal diameter.
Variables including “diameter_a” refer to apical diameter.
Variables including only “diameter” refer to very short shoots, for which it was considered that the basal and apical diameters would be identical, so that just one of them was recorded.
When the one among diameter, diameter_b and diameter_a property is missing for one growth unit, it can be found as the apical/basal diameter of the preceding/successive growth unit.

What the 4 variables for leaf area (leaf_area_2018, leaf_area_2018b, leaf_area_2019, leaf_area_2019b) represent?
The year (2018 or 2019) contained into the variable name refer the year for which the leaf area estimate was performed.
Values under the variables named like “leaf_area_yyyy” refer to estimates of the leaf area including the shoot alone, while variables named like “leaf_area_yyyyb” include the leaf surface of a bourse (leaf_area_yyyyb).

Why “nb_leaves”, “nb_flowers”, “nb_fruits”, “fruit_drops” are only occasionally present?
These variables were only monitored in up to 10, possibly terminal, floral units per tree, when available, in each year 2018 and 2019. The first three variables were monitored during flowering and after fruit set, while “fruit_drops” was monitored in September, close to fruit harvest.

Generally, first order branches (+B) have associated the properties: diameter, length, azimuth, angle and leaf area. However diameter, length, azimuth and angle are not always present, why?
Diameters are not give when <= 3mm.
Angle is not given for very small branches, as it was hard to correctly measure with the protractor.
Azimuth is not measured in some first order branches (+B) from 2019, when these occur to grow height in the canopy, making it impossible to measure them accurately, without importantly bending the tree structure.

May a branching element be longer in 2018 than in 2019?
This is occasionally possible, as a consequence of branch breaking due to mechanical accidents during field operations.

There is occasionally a star “*” symbol in the mtgs. What does that means?
It stands for the “same element as above” and expresses one metamer that is observed repeatedly.

The P scale is populated by a leaf area for both the leaf_area_2018 and leaf_area_2019 properties. What they represent?
The leaf_area_2018 property for the element P1 is the sum of the values stored under the leaf_area_2018 property for all elements having Date equal to 2018. Similarly the leaf_area_2019 property for the element P1 is the sum of the values stored under the leaf_area_2018 property for all elements having Date equal to 2019.

Branching (+B) growth units originating in 2017 contain values for leaf_area_2018 and leaf_area_2019. How is that these elements from 2017 have a leaf area?
The growth units from 2017 that originate from the main axis store leaf area properties for both years 2018 and 2019. These correspond to the sum of the leaf area properties from the same years (2018 or 2019) carried by the whole branching structure originated from the main axis, of which +B is the first element.

Supporting Information - Multiscale organization of plant entities

MTG Scale	Plant Entities	Symbol	Relation of inclusion	Angle (°)	Azimuth (°)	Basal diameter (mm)	Length (cm)	Leaf area (cm^2)	Type of bud fate	Nb leaves	Nb flowers	Nb fruits	Fruit drops
	Tree population		Tree, trunk, branch, A, B, C, D, E, S										
P	Tree		Trunk, branch, A, B, C, D, E, S					x					
GU	1st order GU	A	S			x	x	x	x	x	x	x	x
GU	2nd order GU	B	S	x	x	x	x	x	x	x	x	x	x
GU	3rd order GU	C	S			x	x	x	x	x	x	x	x
GU	4th order GU	D	S			x	x	x	x	x	x	x	x
GU	5th order GU	E	S			x	x	x	x	x	x	x	x
S	Internode/group of internodes	S	S										
